# INCOME-BASED POVERTY AND MATERIAL HARDSHIP PREDICT REDUCED COGNITIVE PERFORMANCE IN OLDER AMERICAN ADULTS

**DOI:** 10.1093/geroni/igae098.4221

**Published:** 2024-12-31

**Authors:** George Daniel, Cedric Williams, Aneesah Lawrence, Kiera Buckley, Delaney Leonard, Darren Bernal, Olivia Bailey, Denee Mwendwa

**Affiliations:** Howard University, Washington, District of Columbia, United States; Howard University, Howard University, District of Columbia, United States; Howard University, Washington, District of Columbia, United States; Howard University, Howard University, District of Columbia, United States; Howard University, Washington, District of Columbia, United States; Howard University, Washington, District of Columbia, United States; Howard University, Washington, District of Columbia, United States; Howard University, Washington, District of Columbia, United States

## Abstract

**Background:**

Research has demonstrated a deleterious impact of lower socioeconomic status (SES) on cognitive functioning. SES has primarily been conceptualized and defined in research as education, income, and/or wealth. This study extends our understanding of the association between SES on cognitive functioning by examining income-based poverty and material hardship as proxies for SES. An additional aim investigated the moderating effects of race and sex on these relationships.

**Methods:**

Participants (n=10,974) in the Health and Retirement Study (HRS) completed sociodemographic and neuropsychological assessments. Analyses determined demographic and health differences by poverty-based income. Subgroup analyses investigated the influence of race and sex. Linear regression determined the impact of income-based poverty and material hardship on cognitive functioning, while controlling for demographic and other covariates.

**Results:**

Mean age was 74 years old. Sample characteristics were 15% Black, 10% Hispanic, and 58% female. Those below the income-based poverty threshold (n=960) were younger, Black, less wealthy, less educated, and unhealthier. Income-based poverty was the strongest predictor of poorer cognitive functioning (B = -1.65, p < 0.001), and this relationship was stronger in Blacks and women. Indicators of material hardship (e.g., food scarcity, rationing medication, and difficulty paying bills) also predicted poorer cognitive functioning.

**Discussion:**

Income-based poverty and material hardship predicted poorer cognitive functioning. Novel measures of SES may help to provide further insight into the negative impact SES has on cognitive functioning, particularly in Blacks and women. Future studies should focus on multidimensional poverty and its implications on long-term cognitive functioning and in older adults.

